# Supplementary material for: What was the impact of the first wave of COVID-19 on the delivery of care to children and adults with congenital heart disease? A qualitative study using online forums
Source: BMJ Open. 2021 Sep 30;11(9):e049006. doi: 10.1136/bmjopen-2021-049006 (PMC8487016; doi:10.1136/bmjopen-2021-049006)
Supplement: Author's manuscript [file bmjopen-2021-049006.draft_revisions.pdf]

BMJ Open is committed to open peer review. As part of this commitment we make the peer review history of every article we publish publicly available.

When an article is published we post the peer reviewers' comments and the authors' responses online. We also post the versions of the paper that were used during peer review. These are the versions that the peer review comments apply to.

The versions of the paper that follow are the versions that were submitted during the peer review process. They are not the versions of record or the final published versions. They should not be cited or distributed as the published version of this manuscript.

BMJ Open is an open access journal and the full, final, typeset and author-corrected version of record of the manuscript is available on our site with no access controls, subscription charges or pay-per-view fees (http://bmjopen.bmj.com).

If you have any questions on BMJ Open's open peer review process please email info.bmjopen@bmj.com

The impact of the first wave of COVID-19 on those with lifelong conditions: a case study of congenital heart disease

Journal:	*BMJ Open*
Manuscript ID	bmjopen-2021-049006
Article Type:	Original research
Date Submitted by the Author:	12-Jan-2021
Complete List of Authors:	Wray, Jo; Great Ormond Street Hospital for Children NHS Foundation Trust, Heart and Lung<br>Pagel, Christina; University College London, Clinical Operational Research Unit<br>Chester, Adrian; The Magdi Yacoub Institute<br>Kennedy, Fiona; Barts Health NHS Trust<br>Crowe, Sonya; University College London, Clinical Operational Research Unit, Department of Mathematics
Keywords:	COVID-19, Quality in health care < HEALTH SERVICES ADMINISTRATION & MANAGEMENT, Congenital heart disease < CARDIOLOGY

SCHOLARONE™
Manuscripts

I, the Submitting Author has the right to grant and does grant on behalf of all authors of the Work (as defined in the below author licence), an exclusive licence and/or a non-exclusive licence for contributions from authors who are: i) UK Crown employees; ii) where BMJ has agreed a CC-BY licence shall apply, and/or iii) in accordance with the terms applicable for US Federal Government officers or employees acting as part of their official duties; on a worldwide, perpetual, irrevocable, royalty-free basis to BMJ Publishing Group Ltd ("BMJ") its licensees and where the relevant Journal is co-owned by BMJ to the co-owners of the Journal, to publish the Work in this journal and any other BMJ products and to exploit all rights, as set out in our licence.

The Submitting Author accepts and understands that any supply made under these terms is made by BMJ to the Submitting Author unless you are acting as an employee on behalf of your employer or a postgraduate student of an affiliated institution which is paying any applicable article publishing charge ("APC") for Open Access articles. Where the Submitting Author wishes to make the Work available on an Open Access basis (and intends to pay the relevant APC), the terms of reuse of such Open Access shall be governed by a Creative Commons licence – details of these licences and which Creative Commons licence will apply to this Work are set out in our licence referred to above.

Other than as permitted in any relevant BMJ Author's Self Archiving Policies, I confirm this Work has not been accepted for publication elsewhere, is not being considered for publication elsewhere and does not duplicate material already published. I confirm all authors consent to publication of this Work and authorise the granting of this licence.

For peer review only

The impact of the first wave of COVID-19 on those with lifelong conditions: a case study of congenital heart disease

Jo Wray[1,2], Christina Pagel[3], Adrian H. Chester[4], Fiona Kennedy[5] and Sonya Crowe[3]

[1] Heart and Lung Directorate, Great Ormond Street Hospital for Children NHS Foundation Trust, London, UK

[2] Institute of Cardiovascular Science, University College London, UK

[3] Clinical Operational Research Unit, University College London, UK

[4] The Magdi Yacoub Institute, Heart Science Centre, Harefield, UK

[5] Barts Health NHS Trust, London, UK

Address for correspondence:
Dr Jo Wray
Great Ormond Street Hospital for Children NHS Foundation Trust
London
WC1N 3JH
Email: jo.wray@gosh.nhs.uk

For peer review only

Abstract

Objectives: Globally, health care systems have been stretched to the limit by the COVID-19 pandemic. Significant changes have had to be made to the way in which non-COVID-19 related care has been delivered. Our objective was to understand, from the perspective of patients with a chronic, life-long condition (congenital heart disease, CHD) and their parents/carers, the impact of COVID-19 on the delivery of care, how changes were communicated and whether health care providers should do anything differently in a subsequent wave of COVID-19 infections.

Design and setting: A series of asynchronous discussion forums set up and moderated by three patient charities via their Facebook pages.

Participants: Patients with CHD and parents/carers of patients with CHD.

Main outcome measures: Qualitative responses to questions posted on the discussion forums.

Results: The forums ran over a 6-week period and involved 111 participants. Following thematic analysis of the transcripts, we identified three themes and ten subthemes related to individual condition-related factors, patient-related factors and health professional/centre factors that may have influenced how patients and parents/carers experienced changes to service delivery as a result of COVID-19.

Conclusions: Our findings, whilst collected in relation to patients with CHD, are not necessarily specific to this population and we believe reflect the experiences of many thousands of people with life-long conditions in the UK. Drawing on what participants told us in the discussion forums, we have developed recommendations related to communication, service delivery and support during the pandemic that would, we think, improve patients' experience of care and, potentially, their

For peer review only

outcomes. Although the data were collected specifically in relation to COVID-19, a number of these recommendations are relevant to the wider delivery of care to patients with chronic underlying health conditions and reflect principles of good communication and service delivery.

Key words: COVID-19; underlying health conditions; discussion forums; children; adults; parents/carers

Strengths and limitations of the study

- Asynchronous discussion forums enable data collection without the potential bias associated with research interviews.
- Online discussion forums faciliate those who may not be able to or want to contribute using more traditional methods of data collection to participate in research.
- During a pandemic, with limited opportunity for face to face contact, online discussion forums enable patients and their carers to express their views in a timely manner and offer a viable way of collecting data.
- Forum users may not be representative of the overall congenital heart disease community; they tend to be female and white.
- Patient charities moderated the forums and participants are therefore likely to be those who already engage with a patient charity.

For peer review only

Background

Since late 2019, COVID-19 has spread rapidly around the world, reaching official pandemic status in March 2020.(1) The speed with which the virus has spread and the trail of physical and psychological illness, death and economic hardship have been extensively documented in the medical and everyday press. Vast amounts of resources have been ploughed into researching the transmission, disease trajectory and risk factors associated with COVID-19. Adults with underlying health conditions have been identified as being at increased risk of developing severe and fatal disease, particularly those with pre-existing hypertension and coronary heart disease.(2) In contrast to the adult population, severe COVID-19 infection in children is rare but there is a lack of comprehensive data on how children with underlying health conditions are affected by COVID-19.(3)

Globally, health care systems have been stretched to the limit and significant changes to the way in which non-COVID-19 related care has been delivered have had to be implemented. The periods of lockdown imposed in many countries and the cessation of non-essential face to face patient contact have necessitated rapid adjustments and adaptation to new ways of delivering and receiving care. Concerns have been raised about the impact of these changes in terms of delayed diagnosis of other health conditions,(4) delays in seeking treatment,(5) cancellations of treatment,(6) greater non-adherence to medical therapy (7) as well as increased mental health problems.(8) Whilst health professionals and the media have been vocal about these potential consequences, far less has been heard from the patients and their families who are being directly affected.

Congenital heart disease (CHD) is one example of a chronic, life-long condition with a spectrum of severity from mild to life-threatening. Both paediatric and adult patients typically require regular follow-up with specialist CHD professionals and tests of cardiac function are a cornerstone of follow-up. But, as with other patient groups, services for patients with CHD have seen significant and abrupt changes over the last 9 months. In common with many other underlying health conditions, it

For peer review only

is currently unclear what risk COVID-19 presents to a patient with CHD. As part of a larger study commissioned by the NHS to develop new ways of measuring the quality of CHD services for both children and adults,(9) we set out to understand, from the perspective of patients and parents/carers, the impact of COVID-19 on the delivery of care, how changes were communicated and whether health care providers should do anything differently in a subsequent wave of COVID-19 infections. Our belief was that the learning and recommendations arising from this work would also be generalisable to the larger population of children and adults receiving care for other chronic health conditions.

Methods

Design

A qualitative approach underpinned by an interpretivist framework was used, in which online discussion forums were employed to elicit participant (patient or parent/carer) views.

Patient and public involvement (PPI)

A patient co-researcher (AC) was involved with each stage of the project, including data analysis and revising drafts of the manuscript. AC also led a PPI group set up as part of the larger overarching study (comprising three adults with CHD and one grandparent of a child with CHD), who reviewed the forum questions and the findings prior to submission. The online discussion forums were moderated by three patient organisations, each of which contributed to the content and format of the questions. A summary of the results will be disseminated to all three charities for publication on their website and will also be disseminated to CHD services nationally via the Adult CHD specialist nurse network and NHS England.

Participants and data collection

The Children's Heart Federation, Little Hearts Matter and the Somerville Foundation, all of which are national UK charities dedicated to the support of patients with CHD and their families, facilitated and

For peer review only

moderated one or more closed, anonymous, asynchronous online discussion groups via their Facebook pages, following an approach that we have successfully used in previous work.(10, 11) We specifically chose these three charities because we wanted to collect views across age ranges (parents of younger children, teenagers and adult patients with CHD) and from those with complex and less complex CHD. Questions were developed by the authors and the content and language revised based on feedback from the charity representatives and PPI group. The charities recommended that separate forums should be facilitated for adult patients with CHD, teenage patients with CHD and parents/carers of children and young people with CHD. Each charity advertised the discussion forums on their home web page and potential participants were directed to the charity's Facebook page where they were able to access further information about the purpose of the forum, how it would be facilitated and the governance surrounding it. People interested in participating were asked to provide some basic demographic information (age, gender, ethnicity, name of CHD defect, location of home and specialist service, relationship to the person with CHD, and age of person with CHD (for parents/carers)). Having completed this information, they were directed to the appropriate closed Facebook group, depending on participant group, where they were able to respond to the posted questions. The research team provided each charity with the agreed questions at the start of the process and the charity determined when new questions should be posted or any prompts introduced, based on responses. The forums took place over a 6-week period, from August 2020 to September 2020. Questions were very similar for each participant group and each charity, with small revisions to wording to reflect the respondent group (e.g. patient- or carer-relevant wording). An example of the questions is provided in Table 1.

Data management and analysis

The charities removed any identifying details from the responses and provided the research team with a single transcript for each forum along with summary demographic details for each participant group. The transcripts were thematically analysed independently by four members of the research team (JW, SC, CP, AC). Codes were attached to segments of data, with similar codes grouped to

For peer review only

create themes and subthemes related to the perceived impact of COVID-19 on the provision of services. The research team met to discuss the themes and subthemes and to agree the descriptive names assigned to them. The themes and suggested recommendations were then sent with the transcripts to another member of the research team (FK) to ensure that all data related to the perceived impact of COVID-19 on the delivery of services were represented appropriately in the themes.

Ethical considerations

The Research Ethics Committee confirmed that ethical approval was not required because the forums were managed by the charities. Each charity placed privacy notices on their websites, clarifying that participants' comments would only be visible to other members of the discussion group and the charity forum moderators and that all identifying information would be removed from discussion posts before being sent to the researchers.

Results

Five forums were run across the three charities, with 109 participants in total. Participant demographics are shown in Table 2.

Three themes and ten subthemes related to individual condition-related factors, patient-related factors and health professional/centre factors were identified, shown in the Figure with illustrative quotes from the forums. Although there is clearly overlap between these factors, particularly in relation to communication, they represented a useful way of interpreting the data.

Patient-related factors

For the majority of participants, routine clinics had been cancelled and appointments had been held via phone or video-link. Participants (both parents and patients) were largely accepting of these changes necessitated by the first wave of COVID-19 and considered them appropriate. They

For peer review only

recognised that COVID-19 was new to everyone and that little was known about it initially, so they were mostly accepting of some of the shortcomings in communication. The timing of scheduled appointments was an important factor, with some patients seen just before the lockdown and highlighting that this was '*lucky*'. In contrast, others expressed uncertainty about when they would be seen and this was exacerbated if communication from their specialist centre was poor. Reported concern and/or distress were notable in patients who were newly diagnosed or who were in the process of transferring between centres: *"As I was moving from one hospital to another I had nothing* [information] *as neither hospital took responsibility for me".* Some patients/parents felt that it was their responsibility to recognise signs of deterioration or the onset of problems and to decide when they or their child should be seen. Many people described the challenges of getting information about follow-up arrangements, illustrated by one patient: *"I spent many months going round in circles and being passed from pillar to post".* Participants described feeling anxious and stressed about delays in treatment, diagnosis or identifying any deterioration in their condition. For some this stress was intensified by the loneliness brought about by the enforced isolation. A number of participants talked about safety, both in terms of perceived risks to their health from being in the hospital environment or using public transport as well as the risks of not being seen face to face and getting the necessary tests and/or interventions: *"COVID stopped me going to [hospital] for my consultation. This has its plus points and minus points. The changes under the circumstances were fine because I would have had to have travelled on public transport and it's something that I wasn't willing to do. However, I prefer to go to the hospital as it puts my mind at ease when they can do the necessary tests required".*

Individual condition-related factors

As with many other chronic health conditions, there is a spectrum of both complexity and stability of CHD and these factors seem to be important determinants of how COVID-19 was perceived to impact patients. A number of those with more complex CHD were very well supported by their specialist service as well as local primary and secondary care services. They described receiving

For peer review only

regular phone calls and written information and, where necessary, individual arrangements for tests at local surgeries or hospitals. In contrast, some others reported having no contact from their specialist centre or guidance about whether they needed to shield and frequently felt that they had to chase for information about changes to services and guidance about shielding. However, whilst they wanted information about arrangements, those patients whose conditions were stable generally expressed low levels of concern about their health and the impact on it of any changes to their care. For patients who were unstable or who had developed new symptoms, however, the added uncertainty about how and when they might be seen was particularly stressful: *"It's horrible knowing I have a critical illness and knowing I need surgery but not knowing how bad it is. For 5 months now I've been in limbo and frightened".*

Health care professional/centre factors

Communication was the factor that evidently had the biggest impact on patients and parents and how they perceived COVID-19 to have affected them or their child. There was general consensus that messaging and advice had been inconsistent, with different centres and different professionals offering different advice about the same thing: *"Communication from centres about shielding was very contradictory"*. Participants described variation in the contact they had had with different professionals involved in their care: some specialist centres provided excellent communication, others provided nothing; some primary and secondary health professionals were described as being exemplary (*"New GP…went over and above*") but other patients reported having *"nothing from anyone*". A distinction was also made between general advice and patient or condition specific advice, with the latter generally more difficult to access. Some respondents reported that clinicians, particularly cardiac specialist nurses (CLNs) who knew them/their medical history, were proactive and responsive to their queries and this was valued by patients and parents: *"I have no concerns as I find the CLNs are accessible by phone or email and I'm confident that if I had any issues I would be seen sooner"*. In contrast, others were clearly feeling very unsupported by professionals, particularly some parents who described feeling forgotten about and *"left to our own devices."* What was clear,

For peer review only

however, was the vital role played by charities in providing information and support to patients and their families, despite the acknowledged financial and other pressures that the organisations have been under.

Whilst there was a degree of acceptance and understanding about changes to services during the first wave of COVID-19, participants expressed very different expectations for managing the on-going situation and clearly articulated that, as awareness and knowledge about COVID-19 are increasing all the time, they are likely to be far less understanding and tolerant of poor communication, delays and cancellations. A number of participants expressed concerns about the big backlog of appointments and the likelihood that quite a few patients will have deteriorated, resulting in additional health issues for them and additional input and costs incurred by the NHS: *"I understand it must be very difficult but if we have a second wave I think appointments for those awaiting surgery should go ahead. I understand it's dangerous, however leaving symptomatic patients without an appointment could be catastrophic. And would subsequently put more pressure/expense on the NHS."*

Discussion

During this study we elicited the views of a diagnostically heterogeneous group of patients with CHD and/or their parents about their experiences of changes to their specialist services as a result of COVID-19, how those changes had been communicated and what should happen in any subsequent wave of COVID-19. We identified a number of condition-related, patient-related and health professional/centre related factors that may have influenced how patients and parents/carers experienced changes to service delivery. The importance of clear, consistent communication cannot be over-estimated. A number of patients seemed to be surprised that they had not had any contact from their specialist centre, particularly those with more complex CHD, indicating that their expectations about communication with their specialist team were not met. The findings from this study suggest a somewhat mixed picture: some respondents reported being very satisfied with

arrangements and described excellent communication and care; others reported some positive aspects of care delivery but they also expressed examples where communication, particularly, had been poor or inconsistent; a third group were very dissatisfied and disappointed with the lack of communication and disruption to their care. Some had a clear sense that as non-COVID-19 patients they were not a priority: *"I felt I was being ignored and that unless you were a person with COVID no-one wanted to know*". Parents particularly expressed their concern with their experience, some of whom saw this as extending beyond cardiac-related care: *"In short, children's care in all sectors just stopped and that is awful*". Participants also described examples of good practice, such as the responsiveness of the clinical nurse specialists, the online support groups facilitated by psychologists and other health professionals, and the freely available YouTube educational videos developed by their consultants. One contributory factor to the different patterns of communication may have been regional levels of COVID-19 infection, with those centres in areas with high levels of infection potentially finding it harder to keep up with communication, particularly if staff were redeployed to provide front-line care in other areas.

Limitations

Although we specifically chose a method of data collection to increase the accessibility of the research to potential participants and did achieve good diversity in terms of where participants lived and their specialist centre, participants did not reflect a broad range of ethnic groups or gender. This may be of particular salience in light of the growing body of evidence that people from black Asian and minority ethnic (BAME) groups have been disproportionately affected by COVID-19, including experiencing higher rates of mortality due to COVID-19.(12) Even if this is not shown to be the case for patients with CHD, such knowledge is likely to contribute to higher levels of anxiety in BAME individuals and may drive greater social isolation and disengagement with health care, which is an important consideration for specialist centres and the wider health service. The lack of participation from BAME groups reflects a recognised problem that they are less likely to engage with, and

For peer review only

participate in, research than their white British counterparts (13) and speaks to the need for targeted strategies to involve, recruit and retain BAME individuals in research projects.

Charities (not limited to those who moderated the discussion forums in this research) were identified as having a vital role in providing support and information to patients and families during the first wave of COVID-19 and at times were the *only* perceived source of information and support. This also highlights a bigger issue of inequity as it will only be those patients and families who are willing and able (through familiarity and adequate language and literacy skills as well as internet resources) to access charity resources who will be able to benefit from them. Furthermore, many of those who are excluded from this will also be those who are less well informed and have less awareness of guidance about issues related to COVID-19. In light of the important role that they play, it may also be timely for charities to reflect on how to increase their appeal to, and membership from, BAME and other under-represented communities.

Our findings, whilst collected in relation to patients with CHD and their parents/carers, are not necessarily specific to this population and we believe reflect the experiences of many thousands of people with life-long conditions in the UK. Health care delivery changed significantly during lockdown and beyond, and as with all changes there are lessons to be learned. Drawing on what participants told us in the discussion forums, we have developed a series of recommendations (Table 3) that would, we think, improve patients' experience of care and, potentially, their outcomes. We believe these are applicable to any patients with underlying health conditions and some, particularly those related to communication, would likely reap large benefits for relatively little input. Whilst the data were collected specifically in relation to COVID-19 and the learning has come from patients' experiences of care during the lockdown, a number of these recommendations are relevant to the wider delivery of care to patients with chronic underlying health conditions and reflect principles of good communication and service delivery.

For peer review only

Acknowledgements

The authors thank The Somerville Foundation, Little Hearts Matter and the Children's Heart Federation for recruiting participants and moderating the forums, and for their contributions, together with the PPI group, to the development and review of the forum questions.

JW is supported by the Great Ormond Street Hospital NIHR Biomedical Research Centre.

Funding statement

This study is independent research funded by the National Institute for Health Research (Policy Research Programme, Congenital Heart Audit: Measuring Progress In Outcomes Nationally (CHAMPION), PR-R20-0318-23001). The views expressed in this publication are those of the author(s) and not necessarily those of the NHS, the National Institute for Health Research or the Department of Health and Social Care.

Competing interest statement

None of the authors have any conflicts of interest to declare.

Contributorship statement

JW – contributed to the design of the study and undertook the initial analysis of the transcripts; wrote the initial draft of the manuscript and approved the final version.

CP – contributed to the design of the study and undertook the initial analysis of the transcripts; revised the manuscript and approved the final version.

AC – contributed to the design of the study and undertook the initial analysis of the transcripts; revised the manuscript and approved the final version.

FK – contributed to the design of the study; independently checked that all data related to the perceived impact of COVID-19 on the delivery of services were represented appropriately in the themes; revised the manuscript and approved the final version.

For peer review only

SC – contributed to the design of the study and undertook the initial analysis of the transcripts; revised the manuscript and approved the final version.

Data sharing statement

No additional data available due to the potential for identification of participants from their qualitative comments.

Figure legend

Factors influencing patients'/parents' experiences of the impact of COVID-19 on service delivery and care

For peer review only

References

1. World Health Organization. WHO announces COVID-19 outbreak a pandemic. 2020:21-8.
2. Flaherty GT, Hession P, Liew CH, Lim BCW, Leong TK, Lim V, et al. COVID-19 in adult patients with pre-existing chronic cardiac, respiratory and metabolic disease: a critical literature review with clinical recommendations. Trop Dis Travel Med Vaccines. 2020;6:16.
3. Sinha IP, Harwood R, Semple MG, Hawcutt DB, Thursfield R, Narayan O, et al. COVID-19 infection in children. Lancet Respir Med. 2020;8(5):446-7.
4. Maringe C, Spicer J, Morris M, Purushotham A, Nolte E, Sullivan R, et al. The impact of the COVID-19 pandemic on cancer deaths due to delays in diagnosis in England, UK: a national, population-based, modelling study. Lancet Oncol. 2020;21(8):1023-34.
5. Rabbone I, Schiaffini R, Cherubini V, Maffeis C, Scaramuzza A, Diabetes Study Group of the Italian Society for Pediatric E, et al. Has COVID-19 Delayed the Diagnosis and Worsened the Presentation of Type 1 Diabetes in Children? Diabetes Care. 2020;43(11):2870-2.
6. COVID Surgical Collaborative. Elective surgery cancellations due to the COVID-19 pandemic: global predictive modelling to inform surgical recovery plans. Br J Surg. 2020;107(11):1440-9.
7. Vakirlis E, Bakirtzi K, Papadimitriou I, Vrani F, Sideris N, Lallas A, et al. Treatment adherence in psoriatic patients during COVID-19 pandemic: Real-world data from a tertiary hospital in Greece. J Eur Acad Dermatol Venereol. 2020.
8. Torales J, O'Higgins M, Castaldelli-Maia JM, Ventriglio A. The outbreak of COVID-19 coronavirus and its impact on global mental health. Int J Soc Psychiatry. 2020;66(4):317-20.
9. Crowe S, Chester A, Ambler G, Wray J, Brown K, von Klemperer K, et al. Congenital Heart Audit: Measuring Progress In Outcomes Nationally (CHAMPION) 2019 [Available from: https://fundingawards.nihr.ac.uk/award/PR-R20-0318-23001.
10. Rajagopal V, Brown K, Pagel C, Wray J. Parental understanding of our communication of morbidity associated with paediatric cardiac surgery: a qualitative study. BMJ Paediatr Open. 2020;4(1):e000578.
11. Wray J, Brown K, Tregay J, Crowe S, Knowles R, Bull K, et al. Parents' Experiences of Caring for Their Child at the Time of Discharge After Cardiac Surgery and During the Postdischarge Period: Qualitative Study Using an Online Forum. J Med Internet Res. 2018;20(5):e155.
12. Public Health England. Beyond the data: understanding the impact of COVID-19 on BAME groups. 2020.
13. Smart A, Harrison E. The under-representation of minority ethnic groups in UK medical research. Ethn Health. 2017;22(1):65-82.

For peer review only

Table 1: Questions for the adult patient forums

The questions for the parent/carer and teenager forums were very similar to these, with minor wording changes to reflect those respondent groups (e.g. designed to appeal to teenagers or wording appropriate for carers rather than patients).

1. Since the start of the COVID-19 pandemic, what changes or disruptions have you experienced to your normal care for congenital heart disease?
    - Do you think these changes were appropriate in the circumstances? What did you feel about them?
    - Are you concerned about the impact of any changes on your health?
    - What did the services do well under the circumstances?
2. How were you told about the changes to services as a result of COVID-19?
    - How well were these changes communicated to you? How could this have been done better?
    - Did you have access to the information you needed? Where did you go to find out information (e.g. your consultant, a charity)? How easy was it to understand the information you were given about COVID-19?
3. Looking to the future now:
    - If there is a second wave of the pandemic, should the NHS do anything differently in terms of its services for congenital heart disease compared to the first wave?
    - Which aspects of services that were disrupted are you keen to see back to normal as soon as possible?
    - Are there any changes that you would be keen to see stay even when the pandemic is over, such as telephone or online consultations?

For peer review only

Table 2: Participant characteristics

	Number (%)
Participants: Adults with CHD	82 (75)
Young people with CHD	3 (3)
Parents/carers of adult patients with CHD	2 (2)
Parents/carers of children with CHD	22 (20)
Participant gender: Male	9 (8)
Female	88 (81)
Unknown	12 (11)
Participant age group: <16 years	1 (1)
16-20	2 (2)
21-30	9 (8)
31-40	26 (24)
41-50	28 (26)
51-60	24 (22)
>61 years	7 (6)
Unknown	12 (11)
Age group of person with CHD: 0-1 years	1 (1)
2-5 years	3 (3)
6-10 years	1 (1)
11-15 years	2 (2)
16-18 years	2 (2)
>18 years	82 (75)
Unknown	18 (17)
Participant ethnicity: White	99 (91)
Non-white	0 (0)
Unknown	10 (9)
Location of specialist service: England (North East)	3 (3)
England (North West)	8 (7)
England (Yorkshire and the Humber)	3 (3)
England (East Midlands)	6 (6)
England (West Midlands)	16 (15)
England (East of England)	3 (3)
England (London)	26 (24)
England (South East)	6 (6)
England (South West)	9 (8)
Wales	1 (1)
Scotland	7 (6)
Northern Ireland/other	1 (1)
Unknown	20 (18)
Location of home: England (North East)	3 (3)
England (North West)	12 (11)
England (Yorkshire and the Humber)	5 (5)
England (East Midlands)	5 (5)
England (West Midlands)	16 (15)
England (East of England)	8 (7)
England (London)	8 (7)
England (South East)	13 (12)
England (South West)	16 (15)
Wales	4 (4)
Scotland	8 (7)
Northern Ireland/other	1 (1)

For peer review only

Unknown	10 (9)
Complexity of CHD: Single ventricle condition	21 (19)
Biventricular condition	83 (76)
Unknown	5 (5)

*A number of participants chose not to provide some or any demographic information

For peer review only

Table 3: Recommendations for improving patients' experience of care and, potentially, their outcomes, based on what participants told us in the discussion forums

Although generated from research related to congenital heart disease, we believe that these recommendations are relevant for patients with any underlying health conditions.

**Communication - generic**
• Consistent information from all healthcare providers in relation to condition-specific advice<br>○ Includes all hospitals, GPs, community services etc<br>○ Should be routinely provided to patients with a particular condition, wherever they receive their care<br>• Produce and share information about the latest guidance and recommendations with those around the patient<br>○ Includes, but not limited to, schools, nurseries and employers<br>○ Ensure guidance is condition-specific and accessible to patients, to facilitate sharing
**Communication – patient specific**
• Clear advice and guidance about shielding (personalised to individual rather than generic)<br>○ Provided to all patients via a range of media (email, letter, easy read, text message +/- telephone<br>• Proactive communication with patients via email or telephone<br>○ To check in with them<br>○ To update them about any changes<br>○ Determined by individual patient circumstances and need<br>• Dedicated email address/phone line with answerphone for patients to call with concerns or questions<br>○ Checked and responded to regularly by someone familiar with their individual case<br>○ Provides clear information about how frequently messages are checked and when a response can be expected
**Service delivery**
• Regular updates about services<br>○ Any curtailment of services, estimated delay times, safety precautions being put in place<br>• Greater flexibility for tests being done locally, more remote monitoring<br>• Telehealth for some/quick catch-ups or where face to face is not necessary<br>○ For communication of routine test results<br>○ Intermediate appointments for patients seen very frequently<br>○ Benefits in terms of reducing travel, time efficiency and safety<br>• Face to face where indicated/necessary<br>○ For medical tests<br>○ Where patients have complex needs<br>○ Underpinned by patient choice about how and where their care should be delivered<br>• Protection of specialist services, COVID free beds<br>• Individualised approach to patient care and follow-up<br>○ Tailored to diagnosis<br>○ Dependent on where an individual is in terms of their care pathway – e.g. waiting for a treatment intervention vs requiring routine check-up
**Support**
• Increased access to online support<br>○ Signposting to existing support groups and websites<br>• Provision of access to<br>○ Support meetings<br>○ Videos made by health professionals<br>○ Other resources established in response to COVID-19

For peer review only

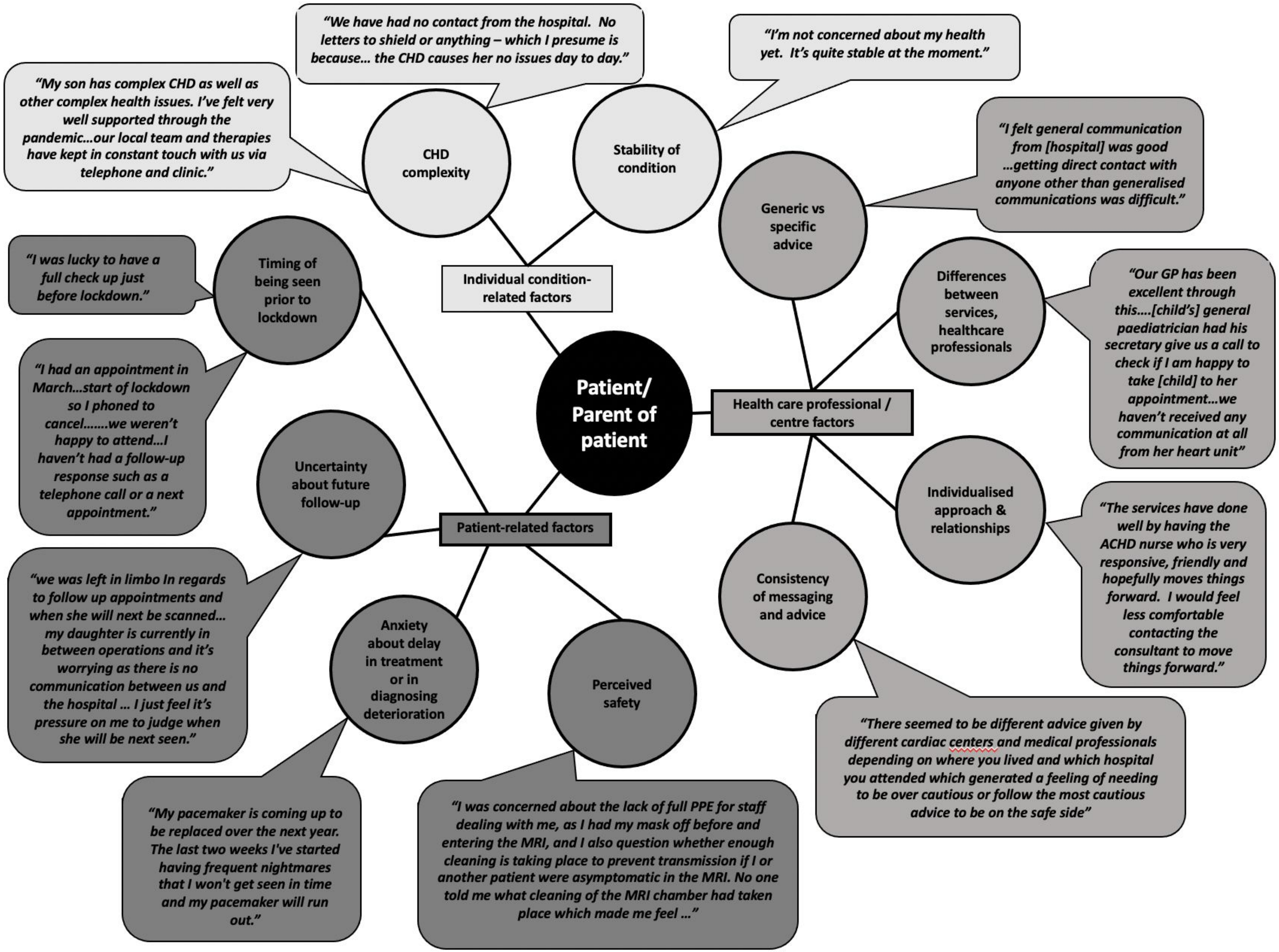
"We have had no contact from the hospital. No letters to shield or anything – which I presume is because… the CHD causes her no issues day to day."
"I'm not concerned about my health yet. It's quite stable at the moment."
"My son has complex CHD as well as other complex health issues. I've felt very well supported through the pandemic…our local team and therapies have kept in constant touch with us via telephone and clinic."
CHD complexity
Stability of condition
"I felt general communication from [hospital] was good …getting direct contact with anyone other than generalised communications was difficult."
Generic vs specific advice
"I was lucky to have a full check up just before lockdown."
Timing of being seen prior to lockdown
Individual condition-related factors
Differences between services, healthcare professionals
"Our GP has been excellent through this….[child's] general paediatrician had his secretary give us a call to check if I am happy to take [child] to her appointment…we haven't received any communication at all from her heart unit"
"I had an appointment in March…start of lockdown so I phoned to cancel…….we weren't happy to attend…I haven't had a follow-up response such as a telephone call or a next appointment."
Patient/ Parent of patient
Health care professional / centre factors
Uncertainty about future follow-up
Patient-related factors
Individualised approach & relationships
"The services have done well by having the ACHD nurse who is very responsive, friendly and hopefully moves things forward. I would feel less comfortable contacting the consultant to move things forward."
Consistency of messaging and advice
"we was left in limbo In regards to follow up appointments and when she will next be scanned… my daughter is currently in between operations and it's worrying as there is no communication between us and the hospital … I just feel it's pressure on me to judge when she will be next seen."
Anxiety about delay in treatment or in diagnosing deterioration
Perceived safety
"There seemed to be different advice given by different cardiac centers and medical professionals depending on where you lived and which hospital you attended which generated a feeling of needing to be over cautious or follow the most cautious advice to be on the safe side"
"My pacemaker is coming up to be replaced over the next year. The last two weeks I've started having frequent nightmares that I won't get seen in time and my pacemaker will run out."
"I was concerned about the lack of full PPE for staff dealing with me, as I had my mask off before and entering the MRI, and I also question whether enough cleaning is taking place to prevent transmission if I or another patient were asymptomatic in the MRI. No one told me what cleaning of the MRI chamber had taken place which made me feel …"

For peer review only

What was the impact of the first wave of COVID-19 on the delivery of care to children and adults with congenital heart disease? A qualitative study using online forums.

Journal:	*BMJ Open*
Manuscript ID	bmjopen-2021-049006.R1
Article Type:	Original research
Date Submitted by the Author:	11-Jun-2021
Complete List of Authors:	Wray, Jo; Great Ormond Street Hospital for Children NHS Foundation Trust, Heart and Lung<br>Pagel, Christina; University College London, Clinical Operational Research Unit<br>Chester, Adrian; The Magdi Yacoub Institute<br>Kennedy, Fiona; Barts Health NHS Trust<br>Crowe, Sonya; University College London, Clinical Operational Research Unit, Department of Mathematics
**Primary Subject Heading**:	Health services research
Secondary Subject Heading:	Communication
Keywords:	COVID-19, Quality in health care < HEALTH SERVICES ADMINISTRATION & MANAGEMENT, Congenital heart disease < CARDIOLOGY

I, the Submitting Author has the right to grant and does grant on behalf of all authors of the Work (as defined in the below author licence), an exclusive licence and/or a non-exclusive licence for contributions from authors who are: i) UK Crown employees; ii) where BMJ has agreed a CC-BY licence shall apply, and/or iii) in accordance with the terms applicable for US Federal Government officers or employees acting as part of their official duties; on a worldwide, perpetual, irrevocable, royalty-free basis to BMJ Publishing Group Ltd ("BMJ") its licensees and where the relevant Journal is co-owned by BMJ to the co-owners of the Journal, to publish the Work in this journal and any other BMJ products and to exploit all rights, as set out in our licence.

The Submitting Author accepts and understands that any supply made under these terms is made by BMJ to the Submitting Author unless you are acting as an employee on behalf of your employer or a postgraduate student of an affiliated institution which is paying any applicable article publishing charge ("APC") for Open Access articles. Where the Submitting Author wishes to make the Work available on an Open Access basis (and intends to pay the relevant APC), the terms of reuse of such Open Access shall be governed by a Creative Commons licence – details of these licences and which Creative Commons licence will apply to this Work are set out in our licence referred to above.

Other than as permitted in any relevant BMJ Author's Self Archiving Policies, I confirm this Work has not been accepted for publication elsewhere, is not being considered for publication elsewhere and does not duplicate material already published. I confirm all authors consent to publication of this Work and authorise the granting of this licence.

For peer review only

What was the impact of the first wave of COVID-19 on the delivery of care to children and adults with congenital heart disease? A qualitative study using online forums.

Jo Wray[1,2], Christina Pagel[3], Adrian H. Chester[4], Fiona Kennedy[5] and Sonya Crowe[3]

[1] Heart and Lung Directorate, Great Ormond Street Hospital for Children NHS Foundation Trust, London, UK

[2] Institute of Cardiovascular Science, University College London, UK

[3] Clinical Operational Research Unit, University College London, UK

[4] The Magdi Yacoub Institute, Heart Science Centre, Harefield, UK

[5] Barts Health NHS Trust, London, UK

Address for correspondence:
Dr Jo Wray
Great Ormond Street Hospital for Children NHS Foundation Trust
London
WC1N 3JH
Email: jo.wray@gosh.nhs.uk

For peer review only

Abstract

Objectives: Globally, health care systems have been stretched to the limit by the COVID-19 pandemic. Significant changes have had to be made to the way in which non-COVID-19 related care has been delivered. Our objective was to understand, from the perspective of patients with a chronic, life-long condition (congenital heart disease, CHD) and their parents/carers, the impact of COVID-19 on the delivery of care, how changes were communicated and whether health care providers should do anything differently in a subsequent wave of COVID-19 infections.

Design and setting: Qualitative study involving a series of asynchronous discussion forums set up and moderated by three patient charities via their Facebook pages.

Participants: Patients with CHD and parents/carers of patients with CHD.

Main outcome measures: Qualitative responses to questions posted on the discussion forums.

Results: The forums ran over a 6-week period and involved 109 participants. Following thematic analysis, we identified three themes and ten subthemes related to individual condition-related factors, patient-related factors and health professional/centre factors that may have influenced how patients and parents/carers experienced changes to service delivery as a result of COVID-19. Specifically, respondents reported high levels of disruption to the delivery of care, inconsistent advice and messaging and variable communication from health professionals, with examples of both excellent and very poor experiences of care reported. Uncertainty about follow-up and factors related to the complexity and stability of their condition contributed to anxiety and stress.

Conclusions: The importance of clear, consistent communication cannot be over-estimated. Our findings, whilst collected in relation to patients with CHD, are not necessarily specific to this

For peer review only

population and we believe reflect the experiences of many thousands of people with life-long conditions in the UK. Recommendations related to communication, service delivery and support during the pandemic may improve patients' experience of care and, potentially, their outcomes.

Key words: COVID-19; underlying health conditions; discussion forums; children; adults; parents/carers

Strengths and limitations of the study

- Asynchronous discussion forums enable data collection without the potential bias associated with research interviews.
- Online discussion forums faciliate those who may not be able to or want to contribute using more traditional methods of data collection to participate in research.
- During a pandemic, with limited opportunity for face to face contact, online discussion forums enable patients and their carers to express their views in a timely manner and offer a viable way of collecting data.
- Forum users may not be representative of the overall congenital heart disease community; they tend to be female and white.
- Patient charities moderated the forums and participants are therefore likely to be those who already engage with a patient charity.

For peer review only

Background

Since late 2019, COVID-19 has spread rapidly around the world, reaching official pandemic status in March 2020.(1) The speed with which the virus has spread and the trail of physical and psychological illness, death and economic hardship have been extensively documented in the medical and everyday press. Vast amounts of resources have been ploughed into researching the transmission, disease trajectory and risk factors associated with COVID-19. Adults with underlying health conditions have been identified as being at increased risk of developing severe and fatal disease, particularly those with pre-existing hypertension and coronary heart disease.(2) In contrast to the adult population, severe COVID-19 infection in children is rare but there is a lack of comprehensive data on how children with underlying health conditions are affected by COVID-19.(3)

Globally, health care systems have been stretched to the limit and significant changes to the way in which non-COVID-19 related care has been delivered have had to be implemented. The periods of lockdown imposed in many countries and the cessation of non-essential face to face patient contact have necessitated rapid adjustments and adaptation to new ways of delivering and receiving care. Concerns have been raised about the impact of these changes in terms of delayed diagnosis of other health conditions,(4) delays in seeking treatment,(5) cancellations of treatment,(6) greater non-adherence to medical therapy (7) as well as increased mental health problems.(8) Whilst health professionals and the media have been vocal about these potential consequences, far less has been heard from the patients and their families who are being directly affected.

Congenital heart disease (CHD) is one example of a chronic, life-long condition with a spectrum of severity from mild to life-threatening. It is the most common birth defect and significant improvements in diagnosis and treatment mean that currently approximately 12 million people live with CHD worldwide.(9) Both paediatric and adult patients typically require regular follow-up with specialist CHD professionals and tests of cardiac function are a cornerstone of follow-up. (10, 11)

For peer review only

But, as with other patient groups, services for patients with CHD have seen significant and abrupt changes since March 2020. In an international survey, patients with CHD and parents/carers reported significant disruption to scheduled cardiac surgery and clinic visits and high levels of psychological stress as a result of the pandemic,(12) supporting findings with other patient cohorts.(13-17) However, *how* and *who* communicates with patients with CHD and/or their carers in relation to COVID-19 has not been explored nor how patients/carers think services should be delivered in the event of a future wave of COVID-19 infection. As part of a larger study commissioned by the NHS to develop new ways of measuring the quality of CHD services for both children and adults,(18) our aim was to understand, from the perspective of patients and parents/carers, the impact of COVID-19 on the delivery of care, how changes were communicated and whether health care providers should do anything differently in a subsequent wave of COVID-19 infections. Our belief was that the learning and recommendations arising from this work would also be generalisable to the larger population of children and adults receiving care for other chronic health conditions.

Methods

Design

A qualitative approach underpinned by an interpretivist framework was used, in which online discussion forums were employed to elicit participant (patient or parent/carer) views.

Patient and public involvement (PPI)

A patient co-researcher (AC) was involved with each stage of the project, including question design, data analysis and revising drafts of the manuscript. AC also led a PPI group set up as part of the larger overarching study (comprising three adults with CHD and one grandparent of a child with CHD), who reviewed the forum questions for content and language and the findings prior to submission. The forum questions and the presentation of the findings were revised based on feedback from the PPI group. The online discussion forums were moderated by three patient

For peer review only

organisations, each of which contributed to the content and format of the questions. A summary of the results has been disseminated to all three charities for publication on their website and has also been disseminated to CHD services nationally via the Adult CHD specialist nurse network and NHS England.

Participants and data collection

The Children's Heart Federation, Little Hearts Matter and the Somerville Foundation, all of which are national UK charities dedicated to the support of patients with CHD and their families, facilitated and moderated one or more closed, anonymous, asynchronous online discussion groups via their Facebook pages, following an approach that we have successfully used in previous work.(19, 20) We specifically chose these three charities because we wanted to collect views across age ranges (parents of younger children, teenagers and adult patients with CHD) and from those with complex and less complex CHD. Questions were developed by the authors and the content and language revised based on feedback from the charity representatives and PPI group. The charities recommended that separate forums should be facilitated for adult patients with CHD, teenage patients with CHD and parents/carers of children and young people with CHD. Each charity advertised the discussion forums on their home web page and potential participants were directed to the charity's Facebook page where they were able to access further information about the purpose of the forum, how it would be facilitated and the governance surrounding it. People interested in participating were asked to provide some basic demographic information (age, gender, ethnicity, CHD defect, location of home and specialist service, relationship to the person with CHD, and age of person with CHD (for parents/carers)). Having completed this information, they were directed to the appropriate closed Facebook group, depending on participant group, where they were able to respond to the posted questions. All patients and parents/carers who wanted to participate were able to do so – there were no exclusion criteria. Participants could join (or leave) the forum at any stage and the recruitment phase lasted for the duration of the forum. The research team provided each charity with the agreed questions at the start of the process and the charity

For peer review only

posted questions one at a time and determined when new questions should be posted or any prompts introduced, based on responses. When no further responses were forthcoming the moderator posted the next question. The forums took place over a 6-week period, from August 2020 to September 2020. Questions were very similar for each participant group and each charity, with small revisions to wording to reflect the respondent group (e.g. patient- or carer-relevant wording). An example of the questions is provided in Table 1.

Data management and analysis

The charities removed any identifying details from the responses and provided the research team with a single transcript for each forum along with summary demographic details for each participant group. The transcripts were thematically analysed independently by four members of the research team (JW, SC, CP, AC), following the staged approach of Braun and Clarke.(21) The first stage of familiarization involved reading the transcripts and making initial notes, before undertaking the second stage of coding. Preliminary codes were attached to segments of data, with similar codes grouped to create themes and subthemes (stage 3) related to the perceived impact of COVID-19 on the provision of services. The research team met to discuss and review the themes and subthemes (stage 4) and to agree the descriptive names assigned to them (stage 5). The themes and suggested recommendations were then sent with the transcripts to another member of the research team (FK) to ensure that all data related to the perceived impact of COVID-19 on the delivery of services were represented appropriately in the themes. Final revisions addressed any identified gaps or omissions.

Ethical considerations

The Research Ethics Committee confirmed that ethical approval was not required because the forums were managed by the charities. Each charity placed privacy notices on their websites, clarifying that participants' comments would only be visible to other members of the discussion group and the charity forum moderators and that all identifying information would be removed from discussion posts before being sent to the researchers.

For peer review only

Results

Five forums were run across the three charities, with 109 participants in total. One charity ran individual forums for each of the three participant groups; one charity had a single forum for adult patients; and the third charity's forum was for parents/carers of patients with CHD. Participant demographics are shown in Table 2.

Three themes and ten subthemes related to patient-related factors, individual condition-related factors and health professional/centre factors were identified, shown in the Figure with illustrative quotes from the forums. Although there is clearly overlap between these factors, particularly in relation to communication, they represented a useful way of interpreting the data.

Patient-related factors

For the majority of participants, routine clinics had been cancelled and appointments had been held via phone or video-link. Participants (both parents and patients) were largely accepting of these changes necessitated by the first wave of COVID-19 and considered them appropriate. They recognised that COVID-19 was new to everyone and that little was known about it initially, so they were mostly tolerant of some of the shortcomings in communication.

The theme of patient-related factors consisted of four subthemes, related to when patients were seen prior to lockdown, uncertainty about future follow-up appointments, anxiety related to any delays in treatment and their perceived safety.

Timing of being seen prior to lockdown

The timing of scheduled appointments was an important factor, with some patients seen just before the lockdown and highlighting that this was '*lucky*'. Some patients described how they had had routine tests in the months before lockdown which reassured them when subsequent appointments

For peer review only

were cancelled or were not face-to-face: *"My appointment was by phone rather than in person. Echo was cancelled but I'd had an MRI in February, thankfully".* In contrast, other patients who were due to be seen at around the time lockdown started decided not to attend and cancelled their appointments, preferring instead to wait.

Uncertainty about future follow-up

Participants expressed uncertainty about when they would be seen and this was exacerbated if communication from their specialist centre was poor. Reported concern and/or distress were notable in patients who were newly diagnosed or who were in the process of transferring between centres: *"As I was moving from one hospital to another I had nothing* [information] *as neither hospital took responsibility for me".* Many people described the challenges of getting information about follow-up arrangements, illustrated by one patient: *"I spent many months going round in circles and being passed from pillar to post".*

Anxiety about delay in treatment or in diagnosing deterioration

Linked to uncertainty about future follow-up was the subtheme of anxiety related to delays and the consequences of these. Participants described feeling anxious and stressed about delays in treatment, diagnosis or identifying any deterioration in their condition. Prior to lockdown a number of patients were waiting for treatment or had planned surgery for later in the year and this was a significant concern: *"The next surgery was 'urgent' and was scheduled but then cancelled due to COVID…My delayed treatment through COVID has been a huge disappointment, cause of stress and who knows what consequences the wait has had".* Some parents talked about the responsibility of monitoring their child for signs of deterioration or the onset of problems and having to decide when their child should be seen: *"My daughter is currently in between operations and it's worrying…they told us to look out for signs such as low sats, energy levels and weight. I just feel it's pressure on me to judge when she will next be seen. I am also worried…it's [COVID-19] going to delay future surgeries, cath labs and MRI".* For some this stress was intensified by the loneliness brought about

by the enforced isolation: *"I've yet to see anyone. I got a shielding letter, that was it. I've found life very lonely and frightening"*. A number of patients also described feeling that, as non-COVID-19 patients, they were not a priority: *"I felt I was being ignored and that unless you were a person with COVID no-one wanted to know*", with potential consequences for their ongoing care and health.

Perceived safety

A number of participants talked about safety, both in terms of perceived risks to their health from being in the hospital environment or using public transport as well as the risks of not being seen face-to-face and getting the necessary tests and/or interventions: *"COVID stopped me going to [hospital] for my consultation. This has its plus points and minus points. The changes under the circumstances were fine because I would have had to have travelled on public transport and it's something that I wasn't willing to do. However, I prefer to go to the hospital as it puts my mind at ease when they can do the necessary tests required".* The need for balance was summed up by one participant: *"Things have to continue but in the safest way possible for all"*.

Individual condition-related factors

As with many other chronic health conditions, there is a spectrum of both complexity and stability of CHD and these two factors seem to be important determinants of how COVID-19 was perceived to impact patients.

CHD complexity

A number of those with more complex CHD were very well supported by their specialist service as well as local primary and secondary care services. They described receiving regular phone calls and written information and, where necessary, individual arrangements for tests at local surgeries or hospitals: *"The practice nurse called every couple of weeks to check we had all we needed…[child] normally has blood tests every 3 months at the local hospital but this couldn't happen so the GP arranged for it to be done at the surgery with one of their nurses…and arranged a time when the*

For peer review only

surgery was empty." In contrast, some others, particularly those with less complex CHD, reported having no contact from their specialist centre and frequently felt that they had to chase for information about changes to services and guidance about shielding: *"I had nothing from anyone. I just found out on my own by looking on the BBC website mainly. I wasn't informed about changes and had to phone [specialist centre] to find out"*.

Stability of condition

Although they wanted information about arrangements, those patients whose conditions were stable generally expressed low levels of concern about their health and the impact on it of any changes to their care: *"My condition is stable and I am well. So for me, COVID-19 hasn't had any impact in terms of cardiac care".* For patients who were unstable or who had developed new symptoms, however, the added uncertainty about how and when they might be seen was particularly stressful: *"It's horrible knowing I have a critical illness and knowing I need surgery but not knowing how bad it is. For 5 months now I've been in limbo and frightened".*

Health care professional/centre factors

The theme of health care professional and centre factors comprised four subthemes related to consistency of messaging and advice, generic versus specific advice, differences between services and individualised approaches and relationships.

Consistency of messaging and advice

Communication was the factor that evidently had the biggest impact on patients and parents and how they perceived COVID-19 to have affected them or their child. There was general consensus that messaging and advice had been inconsistent, with different centres and different professionals offering different advice about the same thing: *"Communication from centres about shielding was very contradictory"* and *"Hospitals were telling people different things. Some hospitals said single ventricle had to shield whereas others said they didn't have to"*. One parent described the advice

For peer review only

she was given about her child: *"Contacted GP to ask if he should shield and was told no…[then] told he should be shielding…"* Some patients also received letters from the government identifying them as extremely vulnerable and that they should be shielding which directly contradicted the advice given to them by their specialist team whereas others were told by their specialist team that they should shield but did not receive any information to that effect from the government. A number of participants commented on the vital role played by charities in providing information and support to patients and their families, although this also highlighted differences between specialist centres in the guidance they were providing: "*There were lots of people getting distressed…because they had heard nothing from their centre when other people had received guidance letters…more consistency in level of service would be useful".*

Generic versus specific advice

Participants made a distinction between general advice and patient or condition specific advice, the latter of which was generally more difficult to access: "*I got a standard email about COVID-19 and my risk. Later on I got personal advice from my consultant and the nurses*". Another patient described how they were initially notified by their hospital about COVID generally and that was followed up by a call from the CHD nurse to make sure they understood and were happy with what they had to do.

Differences between services and healthcare professionals

Participants described variation in the contact they had had with different professionals involved in their care: some described the excellent support they had received from their GP but a complete lack of communication from the specialist centre whereas for others the reverse was true and it was the cardiac team who were supportive in the absence of any contact from their GPs or local teams. As one participant commented, "*not all medically trained individuals are reading off the same hymn sheet*".

For peer review only

Individualised approach and relationships

Some respondents reported that clinicians, particularly cardiac specialist nurses (CLNs) who knew them/their medical history, were proactive and responsive to their queries and this was valued by patients and parents: *"I have no concerns as I find the CLNs are accessible by phone or email and I'm confident that if I had any issues I would be seen sooner"*. Another described how 'their' nurse had been really helpful with advice about COVID, highlighting the value of being able to contact professionals who knew them.

What should health care providers do differently in a subsequent wave of COVID-19?

Whilst there was a degree of acceptance and understanding about changes to services during the first wave of COVID-19, participants expressed very different expectations for managing the on-going situation and clearly articulated that, as awareness and knowledge about COVID-19 are increasing all the time, they are likely to be far less understanding and tolerant of poor communication, delays and cancellations. A number of participants expressed concerns about the big backlog of appointments and the likelihood that quite a few patients will have deteriorated, resulting in additional health issues for them and additional input and costs incurred by the NHS: *"I understand it must be very difficult but if we have a second wave I think appointments for those awaiting surgery should go ahead. I understand it's dangerous, however leaving symptomatic patients without an appointment could be catastrophic. And would subsequently put more pressure/expense on the NHS."*

Based on participants' experiences and responses about what health care professionals should do differently in any subsequent wave of COVID-19, a series of recommendations has been developed in relation to four domains: generic communication, patient-specific communication, service delivery and support (Table 3).

For peer review only

Discussion

The impact of COVID-19 on the delivery of services to patients with CHD in the UK has been significant, with consequences for both patients and their parents/carers in terms of anxiety and stress. Our findings support those of Cousino and colleagues,(12) who also identified high levels of disruption to routine CHD services and resulting effects on mental health, although in contrast to this latter study we did not find a high level of concern expressed about returning to face-to-face appointments. On the contrary, many respondents in our study wanted face-to-face appointments to be reinstated. Although some patients were concerned about their safety in the hospital environment because of the risks associated with COVID-19, as has been reported by parents of children with cancer(22) and asthma,(23) fear about getting COVID-19 was not a dominant theme in our study. Of note, however, is that other studies explicitly asked respondents about their anxiety related to getting COVID-19 and we did not do this.

We were also interested in *how* patients found out about changes to their care and the importance of clear, consistent communication cannot be over-estimated. Lack of consistency in guidance, confused and contradictory messaging and uncertainty characterised many responses, mirroring the national picture in relation to communication about COVID-19 (24) as well as results from studies with other patient groups.(25) A number of patients were surprised that they had not had any contact from their specialist centre, particularly those with more complex CHD who are typically relatively high users of health care, indicating that their expectations about communication with their specialist team were not met, and this mismatch between expectations and reality is likely to have contributed to higher stress levels.(26) The findings from this study suggest a somewhat mixed picture: some respondents reported being very satisfied with arrangements and described excellent communication and care; others reported some positive aspects of care delivery but they also expressed examples where communication, particularly, had been poor or inconsistent; a third group were very dissatisfied and disappointed with the lack of communication and disruption to their care. Participants also described examples of good practice, such as the responsiveness of the

For peer review only

clinical nurse specialists, the online support groups facilitated by psychologists and other health professionals, and the freely available YouTube educational videos developed by their consultants. One contributory factor to the different patterns of communication may have been regional levels of COVID-19 infection, with those centres in areas with high levels of infection potentially finding it harder to keep up with communication, particularly if staff were redeployed to provide front-line care in other areas or were working remotely.

Limitations

Facebook has been used in a variety of ways in numerous studies and remains a dominant player in the social media milieu.(27) Although we specifically chose a method of data collection to increase the accessibility of the research to potential participants and did achieve good diversity in terms of where participants lived and their specialist centre, participants did not reflect a broad range of ethnic groups or gender. This may be of particular salience in light of the growing body of evidence that people from black Asian and minority ethnic (BAME) groups have been disproportionately affected by COVID-19, including experiencing higher rates of mortality due to COVID-19 (28) and heightened levels of anxiety (29). Even if this is not shown to be the case for patients with CHD, such knowledge is likely to contribute to higher levels of anxiety in BAME individuals and may drive greater social isolation and disengagement with health care, which is an important consideration for specialist centres and the wider health service. The lack of participation from BAME groups reflects a recognised problem that they are less likely to engage with, and participate in, research than their white British counterparts (30) and speaks to the need for targeted strategies to involve, recruit and retain BAME individuals in research projects.

Charities (not limited to those who moderated the discussion forums in this research) were identified as having a vital role in providing support and information to patients and families during the first wave of COVID-19 and at times were the *only* perceived source of information and support. This also highlights a bigger issue of inequity as it will only be those patients and families who are

For peer review only

willing and able (through familiarity and adequate language and literacy skills as well as internet resources) to access charity resources who will be able to benefit from them. Furthermore, many of those who are excluded from this will also be those who are less well informed and have less awareness of guidance about issues related to COVID-19. In light of the important role that they play, it may also be timely for charities to reflect on how to increase their appeal to, and membership from, BAME and other under-represented communities.

Our findings, whilst collected in relation to patients with CHD and their parents/carers, are not necessarily specific to this population and we believe reflect the experiences of many thousands of people with life-long conditions in the UK. Health care delivery changed significantly during lockdown and beyond, and as with all changes there are lessons to be learned. The recommendations that have been developed from what participants told us in the discussion forums, would, we think, improve patients' experience of care and, potentially, their outcomes. Monitoring of experiences and outcomes should be routinely undertaken, particularly at a time when patients are more vulnerable, to evaluate the impact of changes to service delivery and support as well as the implications for resource utilisation and to enable further changes to be responsive to patient need. A key element of the recommendations is flexibility and individualisation and our findings clearly demonstrate the diversity in responses to COVID-19, at both a patient and institutional level. We believe the proposed recommendations, monitoring and evaluation are applicable to any patients with underlying health conditions and some, particularly those related to communication, would likely reap large benefits for relatively little input. Whilst the data were collected specifically in relation to COVID-19 and the learning has come from patients' experiences of care during the lockdown, a number of these recommendations are relevant to the wider delivery of care to patients with chronic underlying health conditions and reflect principles of good communication and service delivery.

For peer review only

Acknowledgements

The authors thank The Somerville Foundation, Little Hearts Matter and the Children's Heart Federation for recruiting participants and moderating the forums, and for their contributions, together with the PPI group, to the development and review of the forum questions.

JW is supported by the Great Ormond Street Hospital NIHR Biomedical Research Centre.

Funding statement

This study is independent research funded by the National Institute for Health Research (Policy Research Programme, Congenital Heart Audit: Measuring Progress In Outcomes Nationally (CHAMPION), PR-R20-0318-23001). The views expressed in this publication are those of the author(s) and not necessarily those of the NHS, the National Institute for Health Research or the Department of Health and Social Care.

Competing interest statement

None of the authors have any conflicts of interest to declare.

Contributorship statement

JW – contributed to the design of the study and undertook the initial analysis of the transcripts; wrote the initial draft of the manuscript and approved the final version.

CP – contributed to the design of the study and undertook the initial analysis of the transcripts; revised the manuscript and approved the final version.

AC – contributed to the design of the study and undertook the initial analysis of the transcripts; revised the manuscript and approved the final version.

FK – contributed to the design of the study; independently checked that all data related to the perceived impact of COVID-19 on the delivery of services were represented appropriately in the themes; revised the manuscript and approved the final version.

For peer review only

SC – contributed to the design of the study and undertook the initial analysis of the transcripts; revised the manuscript and approved the final version.

Data sharing statement

No additional data available due to the potential for identification of participants from their qualitative comments.

Figure legend

Factors influencing patients'/parents' experiences of the impact of COVID-19 on service delivery and care

For peer review only

References

1. World Health Organization. WHO announces COVID-19 outbreak a pandemic. 2020:21-8.
2. Flaherty GT, Hession P, Liew CH, Lim BCW, Leong TK, Lim V, et al. COVID-19 in adult patients with pre-existing chronic cardiac, respiratory and metabolic disease: a critical literature review with clinical recommendations. Trop Dis Travel Med Vaccines. 2020;6:16.
3. Sinha IP, Harwood R, Semple MG, Hawcutt DB, Thursfield R, Narayan O, et al. COVID-19 infection in children. Lancet Respir Med. 2020;8(5):446-7.
4. Maringe C, Spicer J, Morris M, Purushotham A, Nolte E, Sullivan R, et al. The impact of the COVID-19 pandemic on cancer deaths due to delays in diagnosis in England, UK: a national, population-based, modelling study. Lancet Oncol. 2020;21(8):1023-34.
5. Rabbone I, Schiaffini R, Cherubini V, Maffeis C, Scaramuzza A, Diabetes Study Group of the Italian Society for Pediatric E, et al. Has COVID-19 Delayed the Diagnosis and Worsened the Presentation of Type 1 Diabetes in Children? Diabetes Care. 2020;43(11):2870-2.
6. COVID Surgical Collaborative. Elective surgery cancellations due to the COVID-19 pandemic: global predictive modelling to inform surgical recovery plans. Br J Surg. 2020;107(11):1440-9.
7. Vakirlis E, Bakirtzi K, Papadimitriou I, Vrani F, Sideris N, Lallas A, et al. Treatment adherence in psoriatic patients during COVID-19 pandemic: Real-world data from a tertiary hospital in Greece. J Eur Acad Dermatol Venereol. 2020.
8. Torales J, O'Higgins M, Castaldelli-Maia JM, Ventriglio A. The outbreak of COVID-19 coronavirus and its impact on global mental health. Int J Soc Psychiatry. 2020;66(4):317-20.
9. Collaborators GBDCHD. Global, regional, and national burden of congenital heart disease, 1990-2017: a systematic analysis for the Global Burden of Disease Study 2017. Lancet Child Adolesc Health. 2020;4(3):185-200.
10. Warnes CA, Williams RG, Bashore TM, Child JS, Connolly HM, Dearani JA, et al. ACC/AHA 2008 Guidelines for the Management of Adults with Congenital Heart Disease: a report of the American College of Cardiology/American Heart Association Task Force on Practice Guidelines (writing committee to develop guidelines on the management of adults with congenital heart disease). Circulation. 2008;118(23):e714-833.
11. Wernovsky G, Rome JJ, Tabbutt S, Rychik J, Cohen MS, Paridon SM, et al. Guidelines for the outpatient management of complex congenital heart disease. Congenit Heart Dis. 2006;1(1-2):10-26.
12. Cousino MK, Pasquali SK, Romano JC, Norris MD, Yu S, Reichle G, et al. Impact of the COVID-19 pandemic on CHD care and emotional wellbeing. Cardiol Young. 2021;31(5):822-8.
13. Philip K, Cumella A, Farrington-Douglas J, Laffan M, Hopkinson N. Respiratory patient experience of measures to reduce risk of COVID-19: findings from a descriptive cross-sectional UK wide survey. BMJ Open. 2020;10(9):e040951.
14. Lou E, Teoh D, Brown K, Blaes A, Holtan SG, Jewett P, et al. Perspectives of cancer patients and their health during the COVID-19 pandemic. PLoS One. 2020;15(10):e0241741.
15. Vogel AC, Schmidt H, Loud S, McBurney R, Mateen FJ. Impact of the COVID-19 pandemic on the health care of >1,000 People living with multiple sclerosis: A cross-sectional study. Mult Scler Relat Disord. 2020;46:102512.
16. Chung CC, Wong WH, Fung JL, Hong Kong RD, Chung BH. Impact of COVID-19 pandemic on patients with rare disease in Hong Kong. Eur J Med Genet. 2020;63(12):104062.

For peer review only

17. Trivisano M, Specchio N, Pietrafusa N, Calabrese C, Ferretti A, Ricci R, et al. Impact of COVID-19 pandemic on pediatric patients with epilepsy - The caregiver perspective. Epilepsy Behav. 2020;113:107527.
18. Crowe S, Chester A, Ambler G, Wray J, Brown K, von Klemperer K, et al. Congenital Heart Audit: Measuring Progress In Outcomes Nationally (CHAMPION) 2019 [Available from: https://fundingawards.nihr.ac.uk/award/PR-R20-0318-23001.
19. Rajagopal V, Brown K, Pagel C, Wray J. Parental understanding of our communication of morbidity associated with paediatric cardiac surgery: a qualitative study. BMJ Paediatr Open. 2020;4(1):e000578.
20. Wray J, Brown K, Tregay J, Crowe S, Knowles R, Bull K, et al. Parents' Experiences of Caring for Their Child at the Time of Discharge After Cardiac Surgery and During the Postdischarge Period: Qualitative Study Using an Online Forum. J Med Internet Res. 2018;20(5):e155.
21. Braun V, Clarke V. Using thematic analysis in psychology. Qualitative research in psychology. 2006;3:77-101.
22. Darlington AE, Morgan JE, Wagland R, Sodergren SC, Culliford D, Gamble A, et al. COVID-19 and children with cancer: Parents' experiences, anxieties and support needs. Pediatr Blood Cancer. 2021;68(2):e28790.
23. Jia Y, Bao J, Yi M, Zhang Z, Wang J, Wang H, et al. Impact of the COVID-19 pandemic on asthma control among children: a qualitative study from caregivers' perspectives and experiences. BMJ Open. 2021;11(5):e046525.
24. Dagnall N, Drinkwater KG, Denovan A, Walsh RS. Bridging the Gap Between UK Government Strategic Narratives and Public Opinion/Behavior: Lessons From COVID-19. Frontiers in Communication. 2020;5:71.
25. Kirkegaard P, Edwards A, Andersen B. Balancing risks: Qualitative study of attitudes, motivations and intentions about attending for mammography during the COVID-19 pandemic. Scand J Public Health. 2021:14034948211002648.
26. Hauken MA, Larsen TM, Holsen I. Meeting reality: young adult cancer survivors' experiences of reentering everyday life after cancer treatment. Cancer Nurs. 2013;36(5):E17-26.
27. Franz D, Marsh HE, Chen JI, Teo AR. Using Facebook for Qualitative Research: A Brief Primer. J Med Internet Res. 2019;21(8):e13544.
28. Public Health England. Beyond the data: understanding the impact of COVID-19 on BAME groups. 2020.
29. Gillard S, Dare C, Hardy J, Nyikavaranda P, Rowan Olive R, Shah P, et al. Experiences of living with mental health problems during the COVID-19 pandemic in the UK: a coproduced, participatory qualitative interview study. Soc Psychiatry Psychiatr Epidemiol. 2021.
30. Smart A, Harrison E. The under-representation of minority ethnic groups in UK medical research. Ethn Health. 2017;22(1):65-82.

For peer review only

Table 1: Questions for the adult patient forums

The questions for the parent/carer and teenager forums were very similar to these, with minor wording changes to reflect those respondent groups (e.g. designed to appeal to teenagers or wording appropriate for carers rather than patients).

1. Since the start of the COVID-19 pandemic, what changes or disruptions have you experienced to your normal care for congenital heart disease?
    - Do you think these changes were appropriate in the circumstances? What did you feel about them?
    - Are you concerned about the impact of any changes on your health?
    - What did the services do well under the circumstances?
2. How were you told about the changes to services as a result of COVID-19?
    - How well were these changes communicated to you? How could this have been done better?
    - Did you have access to the information you needed? Where did you go to find out information (e.g. your consultant, a charity)? How easy was it to understand the information you were given about COVID-19?
3. Looking to the future now:
    - If there is a second wave of the pandemic, should the NHS do anything differently in terms of its services for congenital heart disease compared to the first wave?
    - Which aspects of services that were disrupted are you keen to see back to normal as soon as possible?
    - Are there any changes that you would be keen to see stay even when the pandemic is over, such as telephone or online consultations?

For peer review only

Table 2: Participant characteristics

	Number (%)
Participants: Adults with CHD	82 (75)
Young people with CHD	3 (3)
Parents/carers of adult patients with CHD	2 (2)
Parents/carers of children with CHD	22 (20)
Participant gender: Male	9 (8)
Female	88 (81)
Unknown	12 (11)
Participant age group: <16 years	1 (1)
16-20	2 (2)
21-30	9 (8)
31-40	26 (24)
41-50	28 (26)
51-60	24 (22)
>61 years	7 (6)
Unknown	12 (11)
Age group of person with CHD: 0-1 years	1 (1)
2-5 years	3 (3)
6-10 years	1 (1)
11-15 years	2 (2)
16-18 years	2 (2)
>18 years	82 (75)
Unknown	18 (17)
Participant ethnicity: White	99 (91)
Non-white	0 (0)
Unknown	10 (9)
Location of specialist service: England (North East)	3 (3)
England (North West)	8 (7)
England (Yorkshire and the Humber)	3 (3)
England (East Midlands)	6 (6)
England (West Midlands)	16 (15)
England (East of England)	3 (3)
England (London)	26 (24)
England (South East)	6 (6)
England (South West)	9 (8)
Wales	1 (1)
Scotland	7 (6)
Northern Ireland/other	1 (1)
Unknown	20 (18)
Location of home: England (North East)	3 (3)
England (North West)	12 (11)
England (Yorkshire and the Humber)	5 (5)
England (East Midlands)	5 (5)
England (West Midlands)	16 (15)
England (East of England)	8 (7)
England (London)	8 (7)
England (South East)	13 (12)
England (South West)	16 (15)
Wales	4 (4)
Scotland	8 (7)
Northern Ireland/other	1 (1)

For peer review only

Unknown	10 (9)
Complexity of CHD: Single ventricle condition	21 (19)
Biventricular condition	83 (76)
Unknown	5 (5)

*A number of participants chose not to provide some or any demographic information

For peer review only

Table 3: Recommendations for improving patients' experience of care and, potentially, their outcomes, based on what participants told us in the discussion forums

Although generated from research related to congenital heart disease, we believe that these recommendations are relevant for patients with any underlying health conditions.

Communication - generic

- Consistent information from all healthcare providers in relation to condition-specific advice
  - Includes all hospitals, GPs, community services etc
  - Should be routinely provided to patients with a particular condition, wherever they receive their care
- Produce and share information about the latest guidance and recommendations with those around the patient
  - Includes, but not limited to, schools, nurseries and employers
  - Ensure guidance is condition-specific and accessible to patients, to facilitate sharing

Communication – patient specific

- Clear advice and guidance about shielding (personalised to individual rather than generic)
  - Provided to all patients via a range of media (email, letter, easy read, text message +/- telephone
- Proactive communication with patients via email or telephone
  - To check in with them
  - To update them about any changes
  - Determined by individual patient circumstances and need
- Dedicated email address/phone line with answerphone for patients to call with concerns or questions
  - Checked and responded to regularly by someone familiar with their individual case
  - Provides clear information about how frequently messages are checked and when a response can be expected

Service delivery

- Regular updates about services
  - Any curtailment of services, estimated delay times, safety precautions being put in place
- Greater flexibility for tests being done locally, more remote monitoring
- Telehealth for some/quick catch-ups or where face to face is not necessary
  - For communication of routine test results
  - Intermediate appointments for patients seen very frequently
  - Benefits in terms of reducing travel, time efficiency and safety
- Face to face where indicated/necessary
  - For medical tests
  - Where patients have complex needs
  - Underpinned by patient choice about how and where their care should be delivered
- Protection of specialist services, COVID free beds
- Individualised approach to patient care and follow-up
  - Tailored to diagnosis
  - Dependent on where an individual is in terms of their care pathway – e.g. waiting for a treatment intervention vs requiring routine check-up

Support

- Increased access to online support
  - Signposting to existing support groups and websites
- Provision of access to
  - Support meetings
  - Videos made by health professionals
  - Other resources established in response to COVID-19

For peer review only

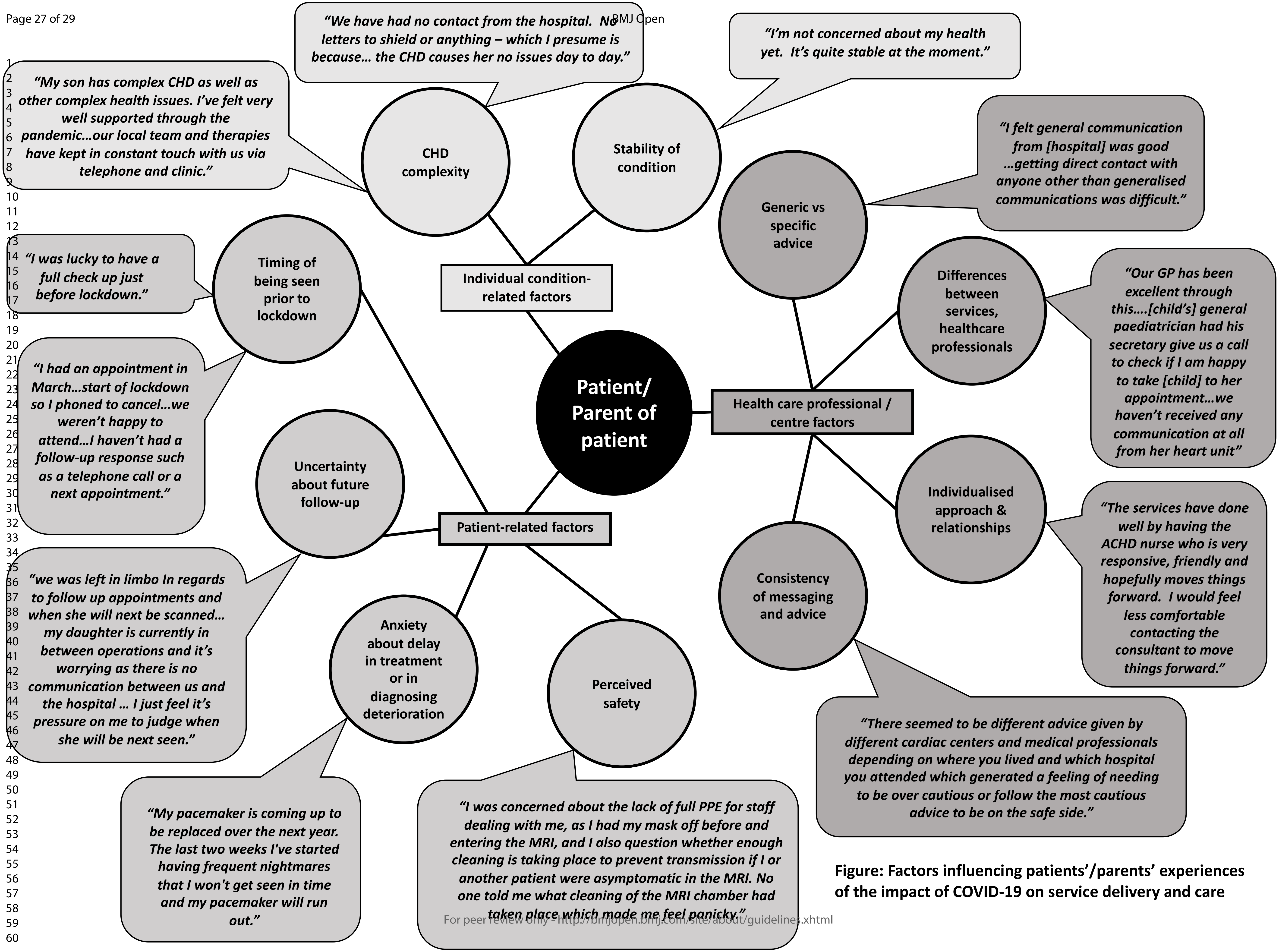

Figure: Factors influencing patients'/parents' experiences of the impact of COVID-19 on service delivery and care

Standards for Reporting Qualitative Research (SRQR)*

http://www.equator-network.org/reporting-guidelines/srqr/

Title and abstract

	Page/line no(s).
**Title** - Concise description of the nature and topic of the study Identifying the study as qualitative or indicating the approach (e.g., ethnography, grounded theory) or data collection methods (e.g., interview, focus group) is recommended	1
**Abstract** - Summary of key elements of the study using the abstract format of the intended publication; typically includes background, purpose, methods, results, and conclusions	2-3

Introduction

**Problem formulation** - Description and significance of the problem/phenomenon studied; review of relevant theory and empirical work; problem statement	4-5
**Purpose or research question** - Purpose of the study and specific objectives or questions	5

Methods

**Qualitative approach and research paradigm** - Qualitative approach (e.g., ethnography, grounded theory, case study, phenomenology, narrative research) and guiding theory if appropriate; identifying the research paradigm (e.g., postpositivist, constructivist/ interpretivist) is also recommended; rationale**	5
**Researcher characteristics and reflexivity** - Researchers' characteristics that may influence the research, including personal attributes, qualifications/experience, relationship with participants, assumptions, and/or presuppositions; potential or actual interaction between researchers' characteristics and the research questions, approach, methods, results, and/or transferability	5 (in relation to patient co-researcher) The research team had no direct contact with any participants
**Context** - Setting/site and salient contextual factors; rationale**	6
**Sampling strategy** - How and why research participants, documents, or events were selected; criteria for deciding when no further sampling was necessary (e.g., sampling saturation); rationale**	6-7
**Ethical issues pertaining to human subjects** - Documentation of approval by an appropriate ethics review board and participant consent, or explanation for lack thereof; other confidentiality and data security issues	7
**Data collection methods** - Types of data collected; details of data collection procedures including (as appropriate) start and stop dates of data collection and analysis, iterative process, triangulation of sources/methods, and modification of procedures in response to evolving study findings; rationale**	6-7

For peer review only - http://bmjopen.bmj.com/site/about/guidelines.xhtml

**Data collection instruments and technologies** - Description of instruments (e.g., interview guides, questionnaires) and devices (e.g., audio recorders) used for data collection; if/how the instrument(s) changed over the course of the study	7, 21
**Units of study** - Number and relevant characteristics of participants, documents, or events included in the study; level of participation (could be reported in results)	8, 22-23
**Data processing** - Methods for processing data prior to and during analysis, including transcription, data entry, data management and security, verification of data integrity, data coding, and anonymization/de-identification of excerpts	7
**Data analysis** - Process by which inferences, themes, etc., were identified and developed, including the researchers involved in data analysis; usually references a specific paradigm or approach; rationale**	7
**Techniques to enhance trustworthiness** - Techniques to enhance trustworthiness and credibility of data analysis (e.g., member checking, audit trail, triangulation); rationale**	7

Results/findings

**Synthesis and interpretation** - Main findings (e.g., interpretations, inferences, and themes); might include development of a theory or model, or integration with prior research or theory	8-13
**Links to empirical data** - Evidence (e.g., quotes, field notes, text excerpts, photographs) to substantiate analytic findings	8-13 + figure

Discussion

**Integration with prior work, implications, transferability, and contribution(s) to the field** - Short summary of main findings; explanation of how findings and conclusions connect to, support, elaborate on, or challenge conclusions of earlier scholarship; discussion of scope of application/generalizability; identification of unique contribution(s) to scholarship in a discipline or field	14-16
**Limitations** - Trustworthiness and limitations of findings	15

Other

**Conflicts of interest** - Potential sources of influence or perceived influence on study conduct and conclusions; how these were managed	17
**Funding** - Sources of funding and other support; role of funders in data collection, interpretation, and reporting	17

*The authors created the SRQR by searching the literature to identify guidelines, reporting standards, and critical appraisal criteria for qualitative research; reviewing the reference lists of retrieved sources; and contacting experts to gain feedback. The SRQR aims to improve the transparency of all aspects of qualitative research by providing clear standards for reporting qualitative research.

For peer review only

**The rationale should briefly discuss the justification for choosing that theory, approach, method, or technique rather than other options available, the assumptions and limitations implicit in those choices, and how those choices influence study conclusions and transferability. As appropriate, the rationale for several items might be discussed together.

Reference:

O'Brien BC, Harris IB, Beckman TJ, Reed DA, Cook DA. **Standards for reporting qualitative research: a synthesis of recommendations.** *Academic Medicine*, Vol. 89, No. 9 / Sept 2014
DOI: 10.1097/ACM.0000000000000388

For peer review only
